# Sleep Quality of Patients with Differentiated Thyroid Cancer

**DOI:** 10.1371/journal.pone.0130634

**Published:** 2015-06-17

**Authors:** Yajing He, Zhaowei Meng, Qiang Jia, Fang Hu, Xianghui He, Jian Tan, Guizhi Zhang, Xue Li, Jianping Zhang, Qing Zhang, Li Liu, Lili Zhao, Jing Li, Yuling Wang, Yumei Qian, Shuling Hou, Hua Liu, Sheng Wang, Renfei Wang, Wei Zheng, Tianpeng Hu, Na Liu, Arun Upadhyaya, Yang Liu

**Affiliations:** 1 Department of Nuclear Medicine, Tianjin Medical University General Hospital, Tianjin, P. R. China; 2 Department of General Surgery, Tianjin Medical University General Hospital, Tianjin, P. R. China; 3 Department of Health Management, Tianjin Medical University General Hospital, Tianjin, P. R. China; University of Rochester, UNITED STATES

## Abstract

**Objective:**

We aimed to measure prevalence of sleep disturbance in patients with differentiated thyroid cancer (DTC) by calculating Pittsburgh Sleep Quality Index (PSQI), and compare these data with patients with benign thyroid nodules or normal participants.

**Methods:**

Three groups of patients participated in this cross-sectional study. In the first group, 162 patients with DTC received total thyroidectomy, and then ^131^I therapy. The second group consisted of 84 patients with benign thyroid nodules, who received partial thyroidectomy. The third group was 78 normal healthy control cases. PSQI was used to assess the sleep quality. Inter-group differences were analyzed by Kruskal-Wallis test or independent samples T test. χ^2^ test was also used to check prevalence differences of poor sleep quality among the groups. Differences of PSQI score and poor sleep quality prevalence before and after ^131^I therapy in the same group of DTC participants were analyzed by paired T test and Mcnemar's test.

**Results:**

Higher PSQI score (7.59 ± 4.21) and higher rate of poor sleep quality (54.32%) were shown in DTC patients than in any other group. After ^131^I therapy, PSQI score and prevalence of poor sleep quality in DTC patients increased significantly to 8.78 ± 4.72 and 70.99%. Then DTC patients were divided into two subgroups based on their metastatic status. DTC patients with metastasis (87/162 cases, 53.70%) had significantly higher PSQI score (10.87 ± 5.18) and higher prevalence of poor sleep quality (79.31%).

**Conclusion:**

DTC patients suffer from sleep disturbance, ^131^I therapy and awareness of metastatic status could worsen sleep problem. Psychological fear of cancer, nuclear medicine therapy and metastasis could be one major underlying reason. Longitude and interventional studies are necessary for further investigations.

## Introduction

Sleep quality is an important factor that affects the quality of life in cancer patients [1]. Poor sleep leads to negative health outcomes, which can often impair patients’ immune systems, lower their cognitive abilities, and cause them unable to perform daily functions [2]. Prevalence of sleep disturbances among patients with cancer is at least twice the rate found in the general population [3]. Patients with cancer are at high risk for poor sleep quality due to the physiological and psychological stressors associated with the disease and its treatments [4–7].

The incidence of thyroid cancer has been increasing rapidly worldwide. Approximately 62980 patients with thyroid cancer are discovered annually in the United States [8]. To minimize the risk of disease recurrence and metastatic spread, adequate surgery and ^131^I ablative therapy are the most important treatments for the management of differentiated thyroid cancer (DTC) [9]. Nevertheless, we do not retrieve any previous literature investigating sleep quality of patients with DTC. Therefore, this study was designed to (1) calculate Pittsburgh Sleep Quality Index (PSQI) score and measure the prevalence of sleep disturbance in patients with DTC, and (2) make comparisons of PSQI score and poor sleep quality prevalence between patients with DTC and patients with benign thyroid nodules, or healthy individuals.

## Participants and Methods

### Participants

Three groups of participants were enrolled in this cross-sectional study. The first sample group included patients with DTC, who received total thyroidectomy in the surgical department of our hospital. These patients received ^131^I ablative therapy approximately one month later in the nuclear medicine department. The second sample group consisted of patients with benign thyroid nodules, who received partial thyroidectomy in the surgical department of our hospital. The third sample group was the normal healthy individuals enrolled from the health management department of our hospital.

Our research was ethically approved by the Institutional Review Board of Tianjin Medical University General Hospital, and this clinical investigation has been conducted according to the principles expressed in the Declaration of Helsinki. Written informed consents were obtained from all participants.

### Group 1

Study participants were post-operative DTC patients who received ^131^I ablative therapy in the nuclear medicine department of our hospital from August 2013 till June 2014. Eligible patients were: (1) interviewed approximately one month after total thyroidectomy, (2) pathological diagnosis of DTC, (3) without mental or psychological disease history, (4) well aware of their disease and (5) literate. Patients with a history of sleep disorders prior to the cancer diagnosis were excluded.

The first measurement of sleep quality of the patients was conducted by trained nurses or residents before ^131^I therapy. All patients received surgery (total thyroidectomy, and cervical lymph node resection in suspected areas) nearly one month before. In this way, we intended to determine the impact of different disease nature and surgery on sleep quality. The second measurement of sleep quality was performed seven days after ^131^I therapy. In this way, we intended to determine the impact of ^131^I ablative therapy and metastatic status acknowledgement on sleep quality.

Treatment protocol was done in accordance with the American thyroid cancer guideline [9]. Briefly, about one month after total thyroidectomy, patients with DTC were given ^131^I for thyroid ablation (approximately 100 mCi). Five days after ^131^I administration, whole body scan and tomographic imaging were performed by using a dual-detector single photon emission computed tomography (SPECT)/CT equipped with high-energy collimators (Discovery NM/CT 670; General Electric Medical Systems, Milwaukee Wisconsin, USA). Clinical assessments (including evaluation of thyroid remnant uptake and whether metastases existed) were conducted after reading the ^131^I scans. Then patients were divided into two subgroups, namely, subgroup 1 DTC with metastases, and subgroup 2 DTC without metastases. Patients were closely followed for at least 6 months. Repeated ^131^I therapies were given with intervals of approximately 6 months, if obvious residue thyroid tissue existed or metastases were detected.

We initially interviewed 179 DTC patients who met our inclusion criteria. Yet, 17 failed to complete the two times sleep quality measurements in our study (9.50% drop-out rate). Therefore, the final number of the first sample was 162 cases. All of the patients provided written informed consent.

### Group 2

Patients in this sample group were post-operative patients with pathological confirmation of benign thyroid nodules. They received partial thyroidectomy in the surgical medicine department of our hospital from August 2013 till March 2014. Eligible patients were: (1) interviewed approximately one month after total thyroidectomy, (2) pathological diagnosis of benign thyroid nodules, (3) without mental or psychological disease history, (4) well aware of their disease and (5) literate. Patients with a history of sleep disorders prior to the thyroid surgery were excluded.

Initially, questionnaires were sent to 92 patients with benign thyroid nodules who met our inclusion criteria. Yet, 8 cases did not have enough information for analysis (8.70% drop-out rate). Therefore, the final number of the second sample was 84 cases. All of them provided written informed consent.

### Group 3

From January 2014 till May 2014, the normal control cohort was enrolled from the health management department of our hospital. Eligible participants were: (1) healthy individuals without any previous diseases, (2) without mental or psychological disease history and (3) literate. Participants with a prior history of sleep disorders were excluded. This cohort included 78 healthy participants, who came to receive annual health checkup. We initially surveyed 82 cases who met our inclusion criteria. Yet, 4 failed to respond with complete information (4.88% drop-out rate).

### Sleep quality measurements

The PSQI score was used to assess sleep quality of the participants based on their sleep experiences during the past week [10]. PSQI is a self-administered questionnaire, proved to be a valid and reliable tool for sleep quality and quantity measurement. PSQI is composed of 19 self-rated questions to calculate 7 dimensions of sleep: subjective sleep quality, sleep latency, sleep duration, habitual sleep efficiency, sleep disturbances, use of sleep medication, and daytime dysfunction. Global scores ranging from 0 to 21 can be obtained from the sum of the 7 dimensional components, and higher scores denote poorer sleep quality. The original authors proposed a cutoff value of 5 in global scores to distinguish poor sleepers (> 5) from good sleepers (≤ 5) [10]. Then, a number of studies have verified that this cutoff value is indeed useful in assessing sleep quality of patients with various kinds of cancer [11–13]. Accordingly, in this study, the patient’s sleep status was considered of poor quality when the PSQI score was >5.

### Statistical analysis

All data were presented as mean ± standard deviation. Statistics were performed with SPSS 17.0 (SPSS Incorporated, Chicago, Illinois, USA). Differences among multiple groups were analyzed by Kruskal—Wallis test. Differences between two groups were analyzed by independent samples T test. χ^2^ test was used to check whether sex had a significant influence on the inter-group differences. χ^2^ test was also used to determine prevalence differences of poor sleep quality among the groups. Differences of PSQI score and poor sleep quality prevalence before and after ^131^I therapy in the same group of DTC participants were analyzed by paired T test and Mcnemar's test. P value not exceeding 0.05 was considered as statistically significant.

## Results

### Impact of disease nature and surgery on sleep quality

Basic data of the participants were listed in Table 1. We did not find any differences in age. And gender did not caused any differences among the groups as well.

**Table 1:** Age and gender composition among the three groups.

		Group 1: patients with DTC* (162 cases)	Group 2: patients with benign thyroid nodules (84 cases)	Group 3: healthy control participants (78 cases)	Statistical analyses: statistical value (P value)
Age (years old)		40.18 ± 13.29	40.77 ± 14.81	39.79 ± 14.59	0.227 (0.893) #
Gender	Male (case number)	38	26	19	1.716 (0.424) $
	Female (case number)	124	58	59	

* DTC = differentiated thyroid cancer;

^#^ analyzed by Kruskal-Wallis test;

^$^ analyzed by χ^2^ test.

By Kruskal-Wallis test, quantitative assessments of the PSQI demonstrated significantly higher score in DTC patients than the other groups (Table 2). Prevalence of poor sleep quality among the three groups were also different. χ^2^ test showed significantly higher rate of poor sleep quality in DTC patients than the other groups (Table 2).

**Table 2:** PSQI* score and prevalence of poor sleep quality among the three groups.

	Group 1: patients with DTC* (162 cases)	Group 2: patients with benign thyroid nodules (84 cases)	Group 3: healthy control participants (78 cases)	Statistical analyses: statistical value (P value)
PSQI* score	7.59 ± 4.21	4.75 ± 2.29	3.96 ± 2.17	57.195 (<0.001) #
Case number for PSQI* > 5	88 (54.32%)	28 (33.33%)	14 (17.95%)	31.169 (<0.001) $
Case number for PSQI* ≤ 5	74	56	64	

* PSQI = Pittsburgh Sleep Quality Index, DTC = differentiated thyroid cancer;

^#^ analyzed by Kruskal-Wallis test;

^$^ analyzed by χ^2^ test.

### Impact of ^131^I therapy and metastatic status acknowledgement on sleep quality

After ^131^I therapy, mean level of PSQI score rose significantly from 7.59 to 8.78 (Table 3). Accordingly, the prevalence of poor sleep quality in DTC patients increased significantly from 54.32% to 70.99% (Table 3). Paired T test and Mcnemar's test revealed significant differences due to ^131^I therapy in the same group of DTC participants.

**Table 3:** PSQI* score and prevalence of poor sleep quality in DTC* patients before and after ^131^I therapy.

	DTC* patients before ^131^I therapy (162 cases)	DTC* patients after ^131^I therapy (162 cases)	Statistical analyses: statistical value (P value)
PSQI* score	7.59 ± 4.21	8.78 ± 4.72	18.139 (<0.001) #
Case number for PSQI* > 5	88 (54.32%)	115 (70.99%)	(0.001) $
Case number for PSQI* ≤ 5	74	47	

* PSQI = Pittsburgh Sleep Quality Index, DTC = differentiated thyroid cancer;

^#^ analyzed by paired T test;

^$^ analyzed by Mcnemar's test.

Furthermore, we divided the patients into two subgroups based on their metastatic status. We found that the awareness of the metastatic status could severely inflicted sleep quality (Table 4). Patients with metastasis (87/162 cases, 53.70% of our cohort) had significantly higher mean level of PSQI score (10.87 ± 5.18), and significantly higher prevalence of poor sleep quality (79.31%).

**Table 4:** PSQI* score and prevalence of poor sleep quality in DTC* patients with or without metastasis.

	DTC* patients with metastasis (87 cases)	DTC* patients without metastasis (75 cases)	Statistical analyses: statistical value (P value)
PSQI* score	10.87 ± 5.18	6.36 ± 2.51	6.886 (<0.001) #
Case number for PSQI* > 5	69 (79.31%)	46 (61.33%)	6.320 (0.015) $
Case number for PSQI* ≤ 5	18	29	

* PSQI = Pittsburgh Sleep Quality Index, DTC = differentiated thyroid cancer;

^#^ analyzed by independent samples T test;

^$^ analyzed by χ^2^ test.

## Discussion

Cancer patients are prone to have an increased risk of sleep disruption. Many cancer patients complain about sleep problems including difficulty in falling asleep, waking frequently during the night, and waking earlier than desired without returning to sleep [14, 15]. Sleep difficulty can induce a variety of detrimental effects. It can cause daytime fatigue, lead to mood disturbances, and impair memory and concentration [15, 16]. In many cases, increasing pain perception and immune-suppression can also happen [17]. Although cancer patients of any type would suffer from sleep problem, the nature of which has not been fully described. It is very likely that cancer patients share some common causes of sleep disturbances, predominantly, the psychological fear about cancer *per se*. Our study sought to prove the concept by describing the sleep problems in patients with DTC, and compare them with patients with benign nodules and normal controls. The only obvious difference between them was the disease nature, and all participants were well aware of their medical status. We demonstrated significantly higher PSQI score and higher rate of poor sleep quality in DTC patients. In addition, surgery seemed to influence sleep as well. We considered that surgery itself and post-surgical side effects (like surgical region pain) inflicted psychological fear and worry on the surgery recipients, which caused them sleep troubles. Patients underwent surgery showed significantly higher PSQI score and higher rate of poor sleep quality than normal control.

It has already been documented that the level of sleep disturbance was significantly elevated at the initiation of radiotherapy or chemotherapy, and maintained elevated over the course of these adjuvant therapies as patients experienced adverse effects of the treatments [12, 15, 18, 19]. It was reported that treatment-induced side effects could lead to anxiety and depression, which affected patients’ sleep qualities [4, 12]. On the one hand, tumor and anti-tumor treatments both increase the production of pro-inflammatory cytokines, which in turn act on the central nervous system and alter rest-activity rhythms and negatively affect sleep [20, 21]. On the other hand, it is also important to keep in mind that psychological fear and worry about these adjuvant therapies could also increase the chances of sleep disturbance. For instance, Tian et al. [12] investigated sleep status of cervical cancer patients during adjuvant therapy. They showed that prevalence rate of poor sleep quality was 52.63% for patients before adjuvant therapy, and 64.50% for patients after adjuvant therapy, the latter was significantly higher than the former. The difference in the PSQI scores before and after adjuvant therapy among cervical cancer patients was also significant. Another report from Gooneratne et al. [13] also noted that frequent use of the neo-adjuvant methods in current lung cancer treatment paradigms increased the prevalence of insomnia. Our results confirmed this point as well. We demonstrated that after ^131^I therapy, mean level of PSQI rose significantly from 7.59 to 8.78, while the prevalence of poor sleep quality in DTC patients increased significantly from 54.32% to 70.99%. ^131^I therapy, being the most representative β radiation nuclear medicine therapy, shares intrinsic similarity with external radiotherapy. The establishment of post-surgical ^131^I treatment for DTC lesions stems from the inherent ability of thyroid cancer cells to concentrate iodine [22]. Nevertheless, psychological fear about nuclear-related issue is very common in the general population, which is even more obvious in the post-surgical DTC patients who experience nuclear medicine treatment themselves. These psychological changes could cause sleep problems of the patients.

Although most DTC patients have relatively good prognosis, this cancer often renders patients no symptoms. As a result, it is usually detected along with cervical lymph nodes involvement in up to 20–50% of patients [23]. However, unlike many other tumor types, the presence of metastatic disease in DTC does not obviate the necessity of surgical excision of the primary tumor. Because metastatic disease will respond to ^131^I therapy, removal of the thyroid and the primary tumor is the most important initial treatment for metastatic disease [9]. After ^131^I treatment, post-therapeutic whole-body scan is conducted to visualize metastases. If abnormal uptake lesions are found, disease stage would be altered, which will affect clinical management in 9–15% of DTC patients [24]. Moreover, SPECT/CT fusion imaging is proved to provide superior lesion localization after ^131^I ablation [25]. Therefore, it is very common for a DTC patient to have metastases to live with. In this study, we adopted SPECT/CT imaging along with whole body scan to assess the ^131^I therapeutic results, as well as the metastatic lesions’ distribution and uptake ability. We found 53.70% of our DTC patients had metastases, either post-surgery pathology proved or post-therapy scan proved. We believe that DTC patients with metastasis suffer and worry psychologically more than otherwise. It was evidently shown that patients with metastases had significantly higher mean level of PSQI, and significantly higher prevalence of poor sleep quality. We did not retrieve any previous paper thoroughly investigating this specific aspect of thyroid cancer.

There are several potential limitations to the present study. First, the assessments of sleep quality in our study were carried out by a different set of interviewers (nurses and residents from three different departments). The difference in investigation skill might increase the difference in sleep quality between groups. However, PSQI is a self-administered scale, and all participants filled out PSQI by themselves. In addition, in order to reduce the confounding factor from the investigators, we have trained our staff beforehand. Therefore, the differences in investigators could only have a minimal impact on sleep measurement. Second, the cross-sectional nature of the investigation did not allow for determination of potential causative role between the studied factors. And we did not assess other aspects of life quality. So we were unable to determine the impact of sleep disturbance on quality of life. Therefore, future research should include a more comprehensive assessment of various risk factors as well as objective measures of sleep. And a longitudinal design should be applied, in which the same individual will be assessed repeatedly at different time points. Third, since sleep problem has been identified, treatments targeting sleep may be important supplemental strategies to optimize well-being of the patients. But, what intervention could be used to solve the sleep trouble? Besides pharmacological therapy [26, 27], behavioral therapy [28, 29], exercise [30], and even Yoga [31] have been tested to be effective to manage sleep dysfunction in cancer patients. We will test these therapeutic methods in our future studies.

## Conclusion

We found that DTC patients suffered from sleep disturbance. Moreover, ^131^I therapy and awareness of metastatic status could worsen their sleep problems. Psychological fear of cancer, nuclear medicine therapy and metastasis could be one major underlying reason for the exacerbation of sleep quality. Further longitude and interventional studies are necessary for investigating the sleep quality aspect of thyroid cancer.
